# Effect of Environmental Tobacco Smoke on Children’s Anxiety and Behavior in Dental Clinics, Jeddah, Saudi Arabia: A Cross-Sectional Study

**DOI:** 10.3390/ijerph18010319

**Published:** 2021-01-04

**Authors:** Heba J. Sabbagh, Ghadeer Sharton, Jumana Almaghrabi, Manal Al-Malik, Mona Hassan Ahmed Hassan, Narmin Helal

**Affiliations:** 1 Department of Pediatric Dentistry, King Abdulaziz University, Jeddah 21589, Saudi Arabia; Nhilal@kau.edu.sa; 2 School of Dentistry, King Abdulaziz University, Jeddah 21589, Saudi Arabia; ghadeersharton@gmail.com (G.S.); jojo_m_1995@hotmail.com (J.A.); 3 Dental Department, King Fahad Armed Forces Hospital, Jeddah 21411, Saudi Arabia; m.almalik@yahoo.com; 4 Department of Biostatistics, High Institute of Public Health, Alexandria University, Alexandria 21561, Egypt; hiph.mhassan@alexu.edu.eg

**Keywords:** dental anxiety, Abeer Anxiety Scale, Franke Behavior Rating Scale, secondhand smoker, children, cooperative patients, dental clinic

## Abstract

Background: Environmental tobacco smoke (ETS) has been linked to behavioral problems, but no study has assessed its relationship with dental anxiety. Therefore, this study’s goal is to assess the relation between ETS and both behavioral problems and dental anxiety among children. Methods: The study sample was collected from two centres in Jeddah from October 2019 to January 2020. Inclusion criteria included healthy 5–16-year-old children having their first dental visit with no emergency complaint. The questionnaire including general information, ETS exposure, the child’s anxiety using the Abeer Children Dental Anxiety Scale (ACDAS) and dental behavior using the Frankl Behavioral Rating Scale. Results: Of 500 children, 337 (67.4% response rate) responded to the questionnaire, among whom 201 (59.6%) had been exposed to passive smoking compared to 136 (40.4%) who had not. Exposed children had a statistically significantly greater tendency to develop anxiety (*p* = 0.002) and demonstrate uncooperative behavior (*p* = 0.006). Generalized linear mode and binary regression analyses suggested that ETS has a statistically significant effect on children’s dental anxiety and behavior (*p* < 0.05). Conclusions: Children exposed to ETS demonstrated statistically significantly higher anxiety levels and uncooperative behavior in the dental clinic compared to those who were not exposed.

## 1. Introduction

Dental anxiety occurs often in children. It is described as a psychological feature or emotional response to a stimulus or experience related to a dental treatment. In children, dental anxiety could greatly influence the child’s oral health condition and management. It has been shown to lead to inflated levels of unhealthy and extracted teeth, episodes of dental pain, and a resulting reduction in their oral health quality of life. Poor oral health can also have notable physical consequences for children, such as disturbed sleep, reduced oral intake, and delayed growth and development. In addition, it may also affect their concentration and lead to poor performance in school. Social interactions with peers can even be affected adversely and children can become subject to bullying because of the appearance of their teeth [1].

Studies showed multiple etiological factors related to negative child’s behavior and anxiety in the dental clinics such as cultural background, parental anxiety, pain and discomfort, age, sociodemographic factors, and genetic and previous dental or sibling experience [2,3,4].

In addition, studies have suggested that there is a correlation between children’s environmental tobacco smoke (ETS) exposure and a variety of mental and behavioral problems including anxiety [5,6]. Studies of each of these problems has suggested distinct effects of both pre- and postnatal exposure associated with parental smoking, and the effects seem to be greatest during fetal development and the first several years of life [7,8]. With respect to older children, ETS has been related to mental problems in those aged 8–15 years [9]. 

ETS is the smoke exhaled from burning tobacco products. Known also as second-hand smoking, passive smoking, or involuntary smoking [10], ETS is among the major health problems that threatens human health around the world and is still one of the most common indoor pollutants worldwide that affect children and adults [11]. 

Tobacco smoke contains several toxic carcinogenic chemicals [7]. The major chemical component is nicotine, exposure to which has been proven to have a significant effect on multiple organ systems prenatally, including the nervous, respiratory, and cardiovascular systems [12]. The effect of ETS may differ from one individual to another depending upon the time and duration of exposure. The cumulative lifespan of exposure to tobacco is related to its’ cumulative harmful effect [8,13]. 

Studies have shown different prevalence of tobacco smoking in Saudi Arabia [14]. It ranged from 12.2% to 52% [15]. A recent study reported more than 40% paternal smoking in Saudi Arabia [16]. This high prevalence had many social contexts among our population. The kingdom of Saudi Arabia has been going through many changes in the society, especially in the last decade. Serious bans and fines has been only effective since 2016 [17]. Despite the serious efforts from the Saudi government to reduce the spread of smoking and increase the population awareness of associated negative smoking sequalae, smoking prevalence is still high. In addition, the real effect of tobacco smoking on children could be underestimated because of their high level of ETS exposure especially from their parents [12,18].

Therefore, the objective of this study was to assess the relation between ETS exposure and both behavior and anxiety of healthy children in a dental clinic.

## 2. Materials and Methods

### 2.1. Subjects

The research question for this study is “do exposure to ETS influence children’s behavior in the dental clinic?” To answer this research question, we conducted a cross-sectional study from 1 October 2019 to 30 January 2020, in two dental referral centers in Jeddah, Saudi Arabia, i.e., King Abdul-Aziz University Dental Hospital (KAUDH) and King Fahad Armed Forces Hospital (KFAFH). The KAUDH and KFAFH Research Ethics Committee, # 05-12-19 and #334, respectively, approved the study.

The sample size was calculated using OpenEpi online calculator with 80% power according to Johnson et al.’s (2000) study results [19]. The suggested sample size was 255. We anticipated 40% nonresponse rate and increased the sample size to 500. 

Inclusion criteria included healthy children 6–16 years old, escorted by one of their parents, who had not received emergency dental treatment before, and had one dental visit with a simple dental procedure, such as prophy, fluoride, or fissure sealants. Exclusion criteria included unescorted children, those 16 or older and 5 years or younger, and those who had an emergency or comprehensive dental treatment or were medically compromised. 

### 2.2. Methods

Two interns interviewed parents, children, and dentists to complete a data collection form composed of three sections: Section 1 included general information about the child and sociodemographic data, Section 2 recorded the child’s ETS exposure, and Section 3 his/her dental anxiety and behavior was assessed using the Abeer Children Dental Anxiety Scale (ACDAS) and Frankl Behavioral Rating Scale (FB). The ACDAS is a validated anxiety scale both in English and Arabic that structures for children 6 years and older with reported Cronbach’s alpha (α) of 0.90 [20].

It includes three main components: Part A: the child’s dental assessment (13 questions); Part B: cognitive section (three questions), and Part C: the section that includes the parent–dentist assessment of the child’s behavior (three questions). Part A assesses the child’s self-reported feelings when faced with dental experiences using three faces: Face 1 indicates happy feeling. Face 2 indicates a neutral, fair, and feeling of OK. Face 3 indicates anxious and scared feeling. The child was required to select a face, and each face was given a score from 1 to 3. The summed score for Part A of the ACDAS ranges from 13 to 39, in which a child is considered anxious if his/her score overall is ≥26 [20]. See Supplementary File S1.

In addition, the dentist used the FB to assess the child’s behavior after a simple dental procedure, such as fluoride, prophy, or fissure sealant. The FB scale ranges from definitely negative (−−ve) (refusing treatment, crying loudly, acting fearful, or any other overt evidence of extreme negativism); negative (−ve) (reluctant to accept treatment); positive (+ve) (accepts treatment, but at times cautious), to definitely positive (good rapport with the dentist, interested in the dental procedures, laughing, and enjoying the situation). The FB was grouped into two (−−ve and −ve under uncooperative and +ve and ++ve under cooperative) to simplify the statistical analysis, particularly, the regression analysis.

The questionnaire also included general questions on the child’s and his/her parents’ sociodemographic variables. It divided parental education into high if their education was more than high school and low if their education was high school or less. Family income was divided into low (less than 4000), moderate (from 4000 to 10,000), and high (more than 10,000) Saudi Riyals per month.

The final version of the English and Arabic versions of the questionnaire was validated. Face validity was conducted with 10 mothers. They were asked whether they had any difficulty understanding or answering any of the questions and the questions were modified accordingly. Then five experts validated the questionnaire’s content by calculating the Item-Content Validity Index (I-CVI) and the Scale-Content Validity Index (S-CVI), which sums to 100%. Reliability Statistics for ACDAS using Cronbach’s alpha was 0.961, which showed a good internal consistency.

The dental interns involved in collecting the data were trained and calibrated by assessing the consistency of their data collection after they interviewed a small group of subjects.

### 2.3. Statistical Analysis

SPSS v. 16 (Inc., Chicago, IL, USA) was used for data entry and analysis. Frequencies and percentages were calculated for categorical variables and means for continuous variables. The *t*-test was used to compare group means. Multiple linear regression model was used to assess the most significant factor among the sociodemographic variables and ETS exposure related to the total score of the child’s dental anxiety (dependent factor), according to Part A of the ACDAS. Binary Regression analysis was used to assess the effect of sociodemographic characteristics and ETS exposure (predictors) on the child’s anxiety and behavior according to the FB combined into the two groups (cooperative and uncooperative: dependent factors); the significance level was set at *p* < 0.05.

## 3. Results

The data collection forms were distributed to 500 participants who were enrolled at the time of the study in both hospitals and met the inclusion criteria. Of those, 337 responded for a 67.4% response rate. The age group 6– <8 years constituted almost half of participants (43.6%) followed by the 8– <10 years children who constituted 33.2% of the study sample and 23.1% were 10 years or older. The sample included 159 (47.2%) females, 178 (52.8%) males, 177 (52.5%) and 146 (43.3%) with high school paternal and maternal education, respectively, and 104 (30.9%) with a high family income. In addition, 201 (59.6%) children were exposed to ETS compared to 136 (40.4%) who were not. Among those who were exposed to ETS, 80 (39.8%) were 6– <8 years, 71 (35.3%) were 8– <10 years, 50 (24.9%) were 10 years or more, 107 (53.2%) were males, 94 (46.8%) were females, 104 (51.7%) and 93 (46.3%) of them had fathers and mothers with high education, respectively, and 59 (29.4%) had a high family income. Comparable percentages were observed among the nonexposed with no statistically significant difference between the two groups (*p* > 0.05: See Table 1).

When assessing the relation between ETS and children’s dental anxiety (Part A), children exposed to ETS showed statistically significantly greater mean anxiety score (*p* = 0.024) compared to unexposed children. The distribution of the sample according to dental anxiety (Part A) is shown in Table 2, where all questions showed statistically significant differences between children exposed and not exposed to ETS (*p* < 0.05). 

With respect to Part B of the ACDAS (cognitive), there was a statistically significant relation between children who were worried about losing control in the dental clinic and ETS (*p* = 0.031). For Part C (child assessment), both parents and the dentist rated ETS children statistically significantly more “OK” than “happy” and more “scared” than “happy” among ETS exposed children (*p* = 0.002) and (*p* = 0.021), respectively. In addition, children exposed to ETS showed statistically significantly more uncooperative behavior (*p* = 0.006) compared to unexposed children according to the FB scale (See Table 3).

Moreover, the multiple linear regression model and binary logistic regression analyses were conducted to assess sociodemographic factors and ETS exposure’s effect on children’s dental anxiety with the ACDAS and FB as dependent factors. ETS showed a statistically significant relation to ACDAS Part A (*p* = 0.002); Part B: if the child was worried about losing control (*p* = 0.027, OR = 0.60, 95% CI = [0.37 to 0.94]); Part C: the way the parents and dentist rated the child (*p* = 0.027, OR = 1.77, 95% CI = [1.07 to 2.91]) and (*p* = 0.011, OR =1.92, 95% CI = [1.16 to 3.17]), respectively, and FB (*p* = 0.003, OR = 2.01, 95% CI = [1.26 to 3.21]): see Table 4 and Table 5). 

## 4. Discussion

This study assessed the associations between children’s dental anxiety and environmental tobacco smoke exposure (ETS). Our results supported previous research that has demonstrated that ETS exposure was related to increased rates of behavior problems in general [21,22]. However, the uniqueness of our study consisted of showing ETS’ effect on children’s anxiety and behavior problems in dental clinics. We found that children exposed to ETS exhibited greater anxiety and uncooperative behavior in dental clinics compared to those who were not.

Dental anxiety is a major problem that is related to fewer dental visits and affects the quality of dental treatment patients perceive [23]. Recent studies have shown increased dental anxiety even when all efforts were made to improve methods for dental management [4]. Understanding the etiology and risk factors related to children’s anxiety could help control and improve the quality of children’s dental care in the future.

The prevalence of dental anxiety reported in our study was 59.6%, which was more than that reported by the World Health Organisation (WHO) in 2004, 40% [11], and what has been reported in other studies carried out in Saudi Arabia [20,24]. Fayad et al.’s (2017) study was conducted on 21–50 year old dental patients (51.6%), and Al-Namankany’s (2017) research was conducted on 6–14 year old school girls (47.6%) [20,25]. The increased prevalence in recent years of those who smoke in the presence of children highlights the problem’s importance. It also calls those responsible for children’s healthcare to improve parental and community awareness of passive smoking’s negative effects on children. Moreover, our study’s advantages over previous studies were that it had a larger sample size, was carried out in the dental setting after a simple dental procedure, included children with no previous dental experience, and was conducted on children of both genders.

This study found a statistically significant effect of ETS on children’s anxiety specifically and behavior in response to dental treatment overall. This was supported by several cohort and cross-sectional studies, some of which had large sample sizes, which reported that ETS, as well as mothers’ tobacco use during pregnancy, is associated with general behavior, hyperactivity/inattention, and emotional problems in children [5,6,21,22,25,26,27,28,29,30]. Moreover, a cross-sectional study with a sample size of 2357 Spanish children reported that children who were exposed to ETS at home for 1 h or more per day were associated with a higher frequency of mental disorders and conduct problems [27]. 

Moreover, the study reported that more children were exposed to ETS among mothers with less education (53.7%) compared to those with higher education (46.3). Although this difference was not statistically significant, it was supported by a previous study that reported that ETS affected mothers with lower educational levels to a greater degree (76.7%) [31]. In addition, Kamel et al., (2019) reported that adult females with a lower education had a higher prevalence of dental anxiety that could affect their children’s anxiety in turn [32]. In addition, Soares et al., (2016) reported a negative effect of family income and child’s anxiety, which supported the statistically significant relation in the binary regression analysis between low family income and children’s dental anxiety found in this study [33]. Moreover, although Wu et al., (2018) reported no significant effect of family income, they still reported a family effect on children’s anxiety [34]. 

In Part C of the ACDAS, both parents and dentists rated children nearly equally as happy, OK, or scared. This differs from what was reported in a previous study, which indicated that British parents rated their children behavior more negatively than did teachers [35]. 

This is the first study to measure the relation between ETS exposure and children’s behavioral problems in dental clinics. Generally, most previous studies have used questionnaires the children and parents answered to assess the child’s behavior. However, this study used a questionnaire three people answered: the child, parents, and dentist. Another strength of this study was that all of the children included had no previous dental experience and had received only a simple procedure on the day of their interview. These inclusion criteria strengthened the study’s outcomes, as other research has considered a previous painful dental experience as one of the main risk factors in dental anxiety [36]. 

One limitation of this study was that parental anxiety was not assessed. However, this was avoided to decrease the questionnaire’s length and allow more responses from the children and parents. Moreover, as it is reported in the literature that smoking affects smokers’ behavior and anxiety, parents who smoke are expected to have greater anxiety, which could have affected their children’s anxiety indirectly. Another limitation is that the simple dental procedure carried out on the children during their first dental visit was conducted by several residents who use different techniques to manage behavior. We tried to overcome this limitation by restricting the behavior management techniques that the residents used to tell-show-do and distraction. In addition, the large range in the children’s age could have affected the result, as older children are expected to have less dental anxiety than younger children [32]. However, although not significant, the mean age of the nonexposed group in our study was younger (7.79 ± 2.43) than the exposed group (8.1 ± 2.32) which favored the results of the exposed group. 

The generalizability of this study is limited. Nevertheless, it could be partially generalized as it includes two main referral centers in Jeddah city serving a larger number of Saudi children, i.e., one from the west (KAUDH) and the other from the East (KFAFH). In addition, both hospitals serve different population sectors. Moreover, the gender and the frequency of young age children (less than 12 years) distribution included in the sample are similar to the general Saudi population [37]. However, the frequency distribution of included older children is less than that the general population. This is because adolescence may seek treatment in primary healthcare clinics, with general dentist or in adult dental clinics, rather than pediatric dental clinics [38].

Future research with even greater sample sizes and a questionnaire that describes the duration and time of ETS exposure fully are recommended to elaborate and understand ETS’ effect further. Moreover, healthcare providers and public health services should improve the public’s awareness of ETS’ adverse effects on children to prevent their exposure to it, and improve their quality of health. 

## 5. Conclusions

Children exposed to ETS demonstrated statistically significantly higher anxiety levels and uncooperative behavior in a dental clinic compared to those who were not. Therefore, this study recommends enquiring about children’s ETS exposure as part of assessing and planning their behavior management during dental visits. 

## Figures and Tables

**Table 1:** Sociodemographic characteristics of included sample by environmental tobacco smoking (ETS) exposure.

Variable		ETS		Total n = 337 (%)	X^2^	*p*
		Exposed n = 201 (%)	Not Exposed n = 136 (%)			
**Age (years)**	6–	80 (39.8)	67 (49.3)	147 (43.6)	2.96	0.227
	8–	71 (35.3)	41 (30.1)	112 (33.2)		
	10+	50 (24.9)	28 (20.6)	78 (23.1)		
**Child gender**	Female	94 (46.8)	65 (47.8)	159 (47.2)	0.03	0.853
	Male	107 (53.2)	71 (52.2)	178 (52.8)		
**Paternal education**	Below university	97 (48.3)	63 (46.3)	160 (47.5)	0.12	0.727
	University or higher	104 (51.7)	73 (53.7)	177 (52.5)		
**Maternal education**	Below university	108 (53.7)	83 (61.0)	191 (56.7)	1.76	0.185
	University or higher	93 (46.3)	53 (39.0)	146 (43.3)		
**Family income (SR)**	<3000	34 (16.9)	26 (19.1)	60 (17.8)	1.15	0.564
	3000–10,000	108 (53.7)	65 (47.8)	173 (51.3)		
	>10,000	59 (29.4)	45 (33.1)	104 (30.9)		

X^2^: Chi square.

**Table 2:** Distribution of the study children by their dental anxiety and exposure to environmental tobacco smoking (ETS).

Abeer Children Dental Anxiety Scale (ACDAS)	ETS						*p*
	Exposed n = 201 (%)			Not Exposed n = 136 (%)			
Child Dental Anxiety	Happy	OK	Scared	Happy	OK	Scared	
How do you feel in waiting area?	49.8	19.9	30.3	64.7	14.7	20.6	0.025 *
How do you feel when doctor is wearing a mask?	52.2	14.9	32.8	67.6	16.2	16.2	0.002 *
How do you feel when you are lying down on dental chair?	53.2	19.4	27.4	65.4	18.4	16.2	0.038 *
How do you feel when doctor exams you with mirror?	59.2	8.0	32.8	68.4	19.9	11.8	<0.001 *
How do you feel when you feel strange taste in your mouth?	43.8	17.4	38.8	52.2	28.7	19.1	<0.001 *
How do you feel having a pinch feeling in your gum?	31.3	26.4	42.3	40.4	35.3	24.3	0.003 *
How do you feel when you have anesthesia (numbness in lips or tongue)?	38.8	17.4	43.8	48.5	25.7	25.7	0.003 *
How do you feel when the dentist cleans your teeth with the buzzy electric arm?	44.3	19.4	36.3	51.5	27.2	21.3	0.011 *
How do you feel when hear the sound of the instrument?	34.2	28.6	37.2	44.8	32.8	22.4	0.015 *
How do you feel when you smell the material and the instrument of the dentist?	47.3	23.4	29.4	54.4	27.9	17.6	0.05 *
How did you feel when your tooth was extracted?	31.3	17.9	50.7	42.6	25.0	32.4	0.004 *
How did you feel when the doctor applied a rubber mask to your face?	31.8	35.3	32.8	28.7	50.7	20.6	0.01 *
How did you feel having a pinch feeling in your hand?	31.8	38.8	29.4	29.4	51.5	19.1	0.039 *
Total dental anxiety score, mean (SD)	25.14 (9.63)			22.07 (7.77)			*t* = 2.26
Mean difference (95% CI)	0.226 (0.029, 0.422)						*p* = 0.024 *

* Significant at *p* = 0.05 (Chi square test or *t* test for total anxiety score).

**Table 3:** Distribution of the study children by their cognitive, assessment, and behavior in relation to environmental tobacco smoking (ETS) exposure.

Abeer Children Dental Anxiety Scale (ACDAS)		ETS		*p*
		Exposed n = 201 (%)	Not Exposed n = 136 (%)	
Child cognitive:				
Does the child feel shy in the clinic?	Yes	78 (38.8)	49 (36.0)	0.610
	No	123 (61.2)	87 (64.0)	
Does he feel shy about the way his teeth look?	Yes	92 (45.8)	49 (36.0)	0.075
	No	109 (54.2)	87 (64.0)	
Is he worried about losing control?	Yes	137 (68.2)	77 (56.6)	0.031 *
	No	64 (31.8)	59(43.4)	
Child Assessment:				
Did he have previous dental treatment?	Yes	104 (51.7)	66 (48.5)	0.56
	No	97 (48.3)	70 (51.5)	
What do you expect his behavior to be in his first visit?	Happy	99 (49.3)	58 (42.6)	0.001 *
	OK	34 (16.9)	47 (34.6)	0.002
	Scared	68 (33.8)	31 (22.8)	0.360
How do you rate his behavior in his visit?	Happy	92 (45.8)	75 (55.1)	0.056 ^a^
	OK	37 (18.4)	29 (21.3)	0.89
	Scared	72 (35.8)	32 (23.5)	0.021 *
Frankl Scale	−−ve	58 (28.9)	30 (22.1)	0.048 *^,a^
	−ve	34 (16.9)	13 (9.6)	0.45
	+ve	42 (20.9)	31 (22.8)	0.28
	++ve	67 (33.3)	62 (45.6)	0.042 *
Grouped Frankl Scale **	Cooperative	109 (54.2)	94 (69.1)	0.006 *
	Uncooperative	92 (45.8)	42 (30.9)	

* Significant at *p* = 0.05 (Chi square test). ** −ve and −−ve were grouped under uncooperative; +ve and ++ve were grouped under cooperative. ^a^ Reference groups.

**Table 4:** Multiple linear regression model for Abeer Children Dental Anxiety Scale (ACDAS) Part A by sociodemographic variables and ETS exposure.

Variable	Unstandardized Coefficients	Std. Error	Standardized Coefficients	*t*	*p*
	B		Beta		
Constant	51.533	11.234		4.587	0.000
Child’s age (years)	−1.568	0.822	−0.104	−1.909	0.057
Child’s gender	0.270	3.755	0.004	0.072	0.943
Paternal education	2.240	4.253	0.032	0.527	0.599
Maternal education	1.820	4.409	0.026	0.413	0.680
Family income (SR)	−5.060	2.844	−0.100	−1.779	0.076
ETS exposure	12.163	3.823	0.172	3.182	0.002 *

Dependent variable is total ACDAS score linearly transformed into 0–100 by the formula, transformed value = (actual value − minimum possible value) × 100/possible range. Dummy independent variables: gender (male = 1), paternal/maternal education (university or higher = 1), ETS exposure (exposed = 1). * Significant at *p* = 0.05 ETS: environmental tobacco smoking.

**Table 5:** Multiple binary logistic regression for children’s cognitive, assessment, and behavior on sociodemographic variables and environmental tobacco smoking (ETS) exposure.

Child Cognitive							
Variable		Does the Child Feel Shy in the Clinic?		Does He Feel Shy about the Way His Teeth Look?		Are You Worried about Losing Control?	
		*p*	OR (95% CI)	*p*	OR (95% CI)	*p*	OR (95% CI)
Age (years)	6– ^a^	0.058		0.424		0.086	
	8–	0.365	0.71 (0.34, 1.49)	0.899	1.05 (0.52, 2.1	0.116	1.85 (0.86, 3.99)
	10+	0.468	1.36 (0.6, 1.49)	0.342	1.45 (0.68, 3.11	0.793	1.12 (0.48, 2.58)
**Gender**	Female	0.136	0.70 (0.44, 1.12)	0.315	1.26 (0.81, 1.96)	0.88	1.03 (0.66, 1.64)
	Male						
**Paternal education**	Below university	0.451	1.23 (0.72, 2.12)	0.261	1.34 (0.81, 2.22)	0.834	0.94 (0.55, 1.62)
	University or higher						
**Maternal education**	Below university	0.161	0.67 (0.38, 1.17)	0.537	0.85 (0.51, 1.43)	0.164	0.67 (0.39, 1.17)
	University or higher						
**Family income (SR)**	<3000 ^a^	0.057		0.884		0.843	
	3000–10,000	0.085	0.53 (0.25, 1.09)	0.695	0.87 (0.44, 1.73)	0.777	1.11 (0.53, 2.33)
	>10,000	0.020 *	0.52 (0.30, 0.90)	0.648	0.90 (0.51, 1.48)	0.559	1.17 (0.69, 2.01)
**ETS**	Yes	0.625	0.89 (0.55, 1.43)	0.062	0.65 (0.41, 1.02)	0.027 *	0.60 (0.37, 0.94)
	No						
**Child Assessment**							
		Has your child had previous dental treatment?		What do you expect the child’s behavior to be in his first dental visit? (parents) **		How do you rate his behavior?	
Age (years)	6– ^a^	0.926		0.959		0.682	
	8–	0.756	1.12 (0.55, 2.27)	0.936	0.97 (0.45, 2.08)	0.954	1.02 (0.47, 2.13
	10+	0.934	1.03 (0.48, 2.23)	0.809	0.90 (0.39, 2.07	0.605	0.80 (0.34, 1.87)
**Gender**	Female	0.315	1.26 (0.80, 1.97)	0.936	1.02 (0.63–1.66)	0.963	1.02 (0.63, 1.65)
	Male						
**Paternal education**	Below university	0.24	0.74 (0.44, 1.23)	0.530	0.84 (0.49, 1.45)	0.264	0.73 (0.42–1.27)
	University or higher						
**Maternal education**	Below university	0.384	0.79 (0.47, 1.34)	0.213	1.43 (0.81, 2.52)	0.056	1.74 (0.99–3.05)
	University or higher						
**Family income (SR)**	<3000 ^a^	<0.001 *		0.352		0.168	
	3000–10,000	0.032 *	2.13 (1.06, 4.25)	0.485	1.31 (0.62, 2.79)	0.165	1.70 (0.81, 3.58)
	>10,000	<0.001 *	2.86 (1.7, 4.81)	0.149	1.52 (0.86–2.67)	0.068	1.71 (0.96–3.02)
**ETS**	Yes	0.417	0.83 (0.53, 1.30)	0.027 *	1.77 (1.07, 2.91)	0.011 *	1.92 (1.16, 3.17)
	No						
Frankl Behavior Rating Scale ***							
**Age (years)**	6– ^a^	0.622 0.926					
	8–		1.03 (0.51, 2.11)				
	10+	0.576	0.80 (0.37, 1.75)				
**Gender**	Female Male	0.171	1.37 (0.87, 2.15)				
		
**Paternal education**	Below university	0.434	0.82 (0.49, 1.36)				
	University or higher						
**Maternal education**	Below university	0.193	1.04 (0.84, 2.41)				
	University or higher						
**Family income (SR)**	<3000 ^a^	0.570					
	3000–10000	0.42	1.33 (0.67, 2.65)				
	>10000	0.838	0.95 (0.57, 1.59)				
**ETS**	Yes	0.003 *	2.01 (1.26, 3.21)				
	No						

** Happy and OK answers were grouped under “not scared” and compared with scared; * Significant at *p* = 0.05; ^a^ Reference groups; *** −ve and −−ve were grouped under uncooperative; +ve and ++ve were grouped under cooperative.

## Data Availability

The data presented in this study are available in the article.

## Supplementary Materials

The following are available online at https://www.mdpi.com/1660-4601/18/1/319/s1, File S1: Abeer Children Dental Anxiety Scale (ACDAS).

## References

[1] Morgan A.G., Rodd H.D., Porritt J.M., Baker S.R., Creswell C., Newton T., Williams C., Marshman Z. (2017). Children’s experiences of dental anxiety. Int. J. Paediatr. Dent..

[2] Klingberg G. (2008). Dental anxiety and behaviour management problems in paediatric dentistry@ a review of background factors and diagnostics. Eur. Arch. Paediatr. Dent..

[3] Sathyaprasad S., Lalugol S., George J. (2018). Prevalence of dental anxiety and associated factors among indian children. Pesqui. Bras. Odontopediatria Clínica Integr..

[4] Moustafa S., Ahmed H. (2015). School children dental health, dental fear and anxiety in relation to their parents’ dental anxiety: Comparative study. J. Nurs. Health Sci..

[5] Tiesler C.M., Chen C.M., Sausenthaler S., Herbarth O., Lehmann I., Schaaf B., Kramer U., von Berg A., von Kries R., Wichmann H.E. (2011). Passive smoking and behavioural problems in children: Results from the LISAplus prospective birth cohort study. Environ. Res..

[6] Hamer M., Ford T., Stamatakis E., Dockray S., Batty G.D. (2011). Objectively measured secondhand smoke exposure and mental health in children: Evidence from the Scottish Health Survey. Arch. Pediatr. Adolesc. Med..

[7] Moritsugu K. (2007). The 2006 Report of the Surgeon General: The health consequences of involuntary exposure to tobacco smoke. Am. J. Prev. Med..

[8] DiFranza J.R., Aligne C.A., Weitzman M. (2004). Prenatal and postnatal environmental tobacco smoke exposure and children’s health. Pediatrics.

[9] Bandiera F.C., Richardson A.K., Lee D.J., He J.P., Merikangas K.R. (2011). Secondhand smoke exposure and mental health among children and adolescents. Arch. Pediatr. Adolesc. Med..

[10] Öberg M.J.M., Pruüss-Üstuün A., Schweizer C., Woodward A. (2010). Second-Hand Smoke: Assessing the Environmental Burden of Disease at National and Local Levels.

[11] Öberg M. (2010). Worldwide burden of disease from exposure to second-hand smoke: A retrospective analysis of data from 192 countries. Lancet.

[12] Abbott L.C., Winzer-Serhan U.H. (2012). Smoking during pregnancy: Lessons learned from epidemiological studies and experimental studies using animal models. Crit. Rev. Toxicol..

[13] Coultas D.B., Peake G.T., Samet J.M. (1989). Questionnaire assessment of lifetime and recent exposure to environmental tobacco smoke. Am. J. Epidemiol..

[14] Ali M.T., Bassiony M. (2009). Smoking in Saudi Arabia. Saudi Med. J..

[15] Moradi-Lakeh M., Bcheraoui C.E., Tuffaha M., Daoud F., Saeedi M.A., Basulaiman M., Memish Z., Almazroa M.A., Rabeeah A.A.A., Mokdad A. (2015). Tobacco consumption in the Kingdom of Saudi Arabia, 2013: Findings from a national survey. BMC Public Health.

[16] Sabbagh H., Khogeer L., Hassan M.A., Allaf H.K. (2020). Parental knowledge and attitude regarding e-cigarette use in saudi arabia and the effect of parental smoking: A cross-sectional study. Risk Manag. Healthcare Policy.

[17] Tough Anti-Tobacco Law Comes into Effect Today. http://www.arabnews.com/node/935816/saudi-arabia.

[18] Warren C., Jones N., Peruga A., Chauvin J., Baptiste J.-P., Silva V.C.D., Awa F.E., Tsouros A., Rahman K., Fishburn B. (2008). Global youth tobacco surveillance, 2000–2007. Morb. Mortal. Wkly. Rep. Surveill. Summ..

[19] Sullivan K.M., Soe M.M. (2007). Documentation for Sample Size for a Cross-Sectional, Cohort, or Clinical Trial Studies. https://www.openepi.com/PDFDocs/SSCohortDoc.pdf.

[20] Al-Namankany A., Ashley P., Petrie A. (2012). The development of a dental anxiety scale with a cognitive component for children and adolescents. Pediatr. Dent..

[21] Lin Q., Hou X.Y., Yin X.N., Wen G.M., Sun D., Xian D.X., Fan L., Jiang H., Jing J., Jin Y. (2017). Prenatal exposure to environmental tobacco smoke and hyperactivity behavior in Chinese young children. Int. J. Environ. Res. Public Health.

[22] Leung C.Y., Leung G.M., Schooling C.M. (2015). Early second-hand smoke exposure and child and adolescent mental health: Evidence from Hong Kong’s ‘Children of 1997’ birth cohort. Addiction.

[23] Alsadat F.A., El-Housseiny A.A., Alamoudi N.M., Elderwi D.A., Ainosa A.M., Dardeer F.M. (2018). Dental fear in primary school children and its relation to dental caries. Niger. J. Clin. Pract..

[24] Williams G.M., O’Callaghan M., Najman J.M., Bor W., Andersen M.J., Richards D., U C. (1998). Maternal cigarette smoking and child psychiatric morbidity: A longitudinal study. Pediatrics.

[25] Fayad M.I., Elbieh A., Baig M.N., Alruwaili S.A. (2017). Prevalence of dental anxiety among dental patients in Saudi Arabia. J. Int. Soc. Prev. Community Dent..

[26] Twardella D., Bolte G., Fromme H., Wildner M., von Kries R., Group G.M.E.S. (2010). Exposure to secondhand tobacco smoke and child behaviour—Results from a cross-sectional study among preschool children in Bavaria. Acta Paediatr..

[27] Yang H.S., Lim H., Choi J., Bae S., Kim Y., Kwon H.J., Ha M. (2018). Environmental tobacco smoke exposure at home and attributable problem behaviors in korean children and adolescents for 2012–2014 in a Nationally Representative Survey. J. Korean Med. Sci..

[28] Tanaka K., Miyake Y., Furukawa S., Arakawa M. (2016). Perinatal smoking exposure and behavioral problems in Japanese children aged 5 years: The kyushu okinawa maternal and child health study. Environ. Res..

[29] Moylan S., Gustavson K., Overland S., Karevold E.B., Jacka F.N., Pasco J.A., Berk M. (2015). The impact of maternal smoking during pregnancy on depressive and anxiety behaviors in children: The Norwegian Mother and Child Cohort Study. BMC Med..

[30] Chastang J., Baiz N., Cadwallader J.S., Robert S., Dywer J.L., Charpin D.A., Caillaud D., de Blay F., Raherison C., Lavaud F. (2015). Postnatal environmental tobacco smoke exposure related to behavioral problems in children. PLoS ONE.

[31] Alghamdi A.S. (2016). Socioeconomic determinants of exposure to secondhand smoke among pregnant women. Int. J. Women’s Health Reprod. Sci..

[32] Kamel A.M., Al-Harbi A.S., Al-Otaibi F.M., Al-Qahtani F.A., Al-Garni A.M. (2019). Dental anxiety at riyadh elm university clinics. Saudi J. Oral Sci..

[33] Soares F.C., Lima R.A., Santos Cda F., de Barros M.V., Colares V. (2016). Predictors of dental anxiety in Brazilian 5–7 years old children. Compr. Psychiatry.

[34] Wu L., Gao X. (2018). Children’s dental fear and anxiety: Exploring family related factors. BMC Oral Health.

[35] Niclasen J., Teasdale T.W., Andersen A.M., Skovgaard A.M., Elberling H., Obel C. (2012). Psychometric properties of the danish strength and difficulties questionnaire: The SDQ assessed for more than 70,000 raters in four different cohorts. PLoS ONE.

[36] Dahlander A., Soares F., Grindefjord M., Dahllof G. (2019). Factors associated with dental fear and anxiety in children aged 7 to 9 years. Dent. J..

[37] GAS (2016). Demography Survey 2016 Saudi Arabia. General Authority for Statistics. https://www.stats.gov.sa/en/4522.

[38] AAPD (2020). Adolescent Oral Health Care. The Reference Manual of Pediatric Dentistry. Chic. Ill Am. Acad. Pediatric Dent..

