# Supplementary material for: Effect of Environmental Tobacco Smoke on Children’s Anxiety and Behavior in Dental Clinics, Jeddah, Saudi Arabia: A Cross-Sectional Study
Source: Int J Environ Res Public Health. 2021 Jan 4;18(1):319. doi: 10.3390/ijerph18010319 (PMC7795303; doi:10.3390/ijerph18010319)
Supplement: Supplementary file 1 [file ijerph-18-00319-s001.pdf]

Abeer Children Dental Anxiety Scale (ACDAS)

Date: age: Gender: M / F

A. THE DENTAL PART

I would like you please to tell me how relaxed or scared you feel at the dentist. Please use the scale below **from 1 to 3**, and tick (√) under the face that shows us **how you feel now**.

1=Happy 2=OK 3=Scared

**How do you feel about:**	1	2	3
1. Sitting in the waiting room?			
2. A dentist wearing a mask on his face?			
3. Laying flat on the dental chair?			
4. A dentist checking your teeth with a mirror?			
5. Having a strange taste in your mouth e.g. a filling or gloves?			
6. Having a "pinch" feeling in your gum?			
7. The feeling of numbness (fat lip or tongue)?			
8. A dentist cleaning your teeth by buzzy electric arm that's spraying water?			
9. The sounds that you hear at the dentist?			
10. The smell at the dentist?			
11. Having a tooth taken out?			
12. Wearing a small rubbery mask on your nose to breathe special gas to help you feel comfortable during treatment?			
13. Having a "pinch" feeling on the back of your hand?			

B. THE COGNITIVE PART

14. Do you feel shy at the dentist? **1. Yes 2. No**
15. Do you feel shy because of the way your teeth look? **1. Yes 2. No**
16. Are you worried about losing control at the dentist? **1. Yes 2. No**

C. THE CHILD ASSESSMENT

For legal guardian: 17. Has your child had previous dental treatment? **1. Yes 2. No**

18. How do you expect your child's behaviour today?

1. Happy 2. OK 3. Scared

For Operator: 19. At the end of this visit, what is your rating for the child's behaviour?

1. Happy 2. OK 3. Scared

Scale copyright © 2011 Dr.Abeer Al-Namankany. All rights reserved.
